# Measurement of Diffusion and Segregation in Semiconductor Quantum Dots and Quantum Wells by Transmission Electron Microscopy: A Guide

**DOI:** 10.3390/nano9060872

**Published:** 2019-06-08

**Authors:** Thomas Walther

**Affiliations:** Kroto Centre for High Resolution Imaging & Analysis, Department of Electronic & Electrical Engineering, University of Sheffield, Sheffield S3 7HQ, UK; t.walther@sheffield.ac.uk

**Keywords:** interdiffusion, segregation, quantum dots, quantum wells, electron microscopy

## Abstract

Strategies are discussed to distinguish interdiffusion and segregation and to measure key parameters such as diffusivities and segregation lengths in semiconductor quantum dots and quantum wells by electron microscopy methods. Spectroscopic methods are usually necessary when the materials systems are complex while imaging methods may suffice for binary or simple ternary compounds where atomic intermixing is restricted to one type of sub-lattice. The emphasis on methodology should assist microscopists in evaluating and quantifying signals from electron micrographs and related spectroscopic data. Examples presented include CdS/ZnS core/shell particles and SiGe, InGaAs and InGaN quantum wells.

## 1. Introduction

Semiconductor quantum domain systems include quantum wells (QWs), quantum nanowires (QNWs) and quantum dots (QDs) where charge carriers (electrons and/or holes) are spatially confined so they can only move in either two (QW), one (QNW) or zero (QD) dimensions and the lateral dimensions are so small that they influence the carrier confinement energies. 

Quantum mechanics shows for a type-I QW with finite offset between the conduction band levels of the two adjacent materials that the electron wavefunction is mainly confined to the material with the lower conduction band level, leaking only little into the neighbouring barrier material, and the energy levels of the electron depend both on the potential offset (depth of QW) and the thickness of the intermediate layer (width of the QW).

For the idealised model of an infinitely deep QW of thickness *L*, the electron energy above the conduction band minimum of the well, *E_e_*, is given by quantum mechanics as

$$E_{e}^{w}=\frac{h^{2}n^{2}}{8m_{e}^{w}L^{2}}$$

wherein *h* is Planck’s constant, n∈N is an integer number (*n* = 1 is the ground state) and m* = $m_{e}^{w}$ is the effective electron mass in the well, and so depends strongly on the QW thickness. For finite well depths, *V*, the wavefunction extends from the well into the barrier, and one needs to distinguish for the electron its effective mass in the well, $m_{e}^{w}$ from that in the barrier, $m_{e}^{b}$. Continuity of the wavefunction at the boundary and energy conservation yields:

$$\tan\left(\frac{2\pi^{2}m_{e}^{w}\;E_{e}^{w}L^{2}}{h^{2}}\right)\;=\sqrt{\frac{m_{e}^{b}\left(V-E_{e}^{w}\right)}{m_{e}^{w}\;E_{e}^{w}}}$$

Note that [1] lists interchanged superscripts on the right hand side of this equation. While this equation can only be solved numerically, it is thus clear that any interdiffusion between well and barrier will change the electron’s confinement energy via two related effects that occur simultaneously, namely increase in the width of the quantum well, *L*, and decrease of the confining potential, *V*. For direct bandgap semiconductors, this will influence the emission energy, *E_PL_*, observed in photoluminescence spectroscopy, which is given by

$$E_{PL}=E_{g}^{w}+E_{e}^{w}+E_{hh}^{w}-E_{X}$$

where $E_{g}^{w}$ denotes the bandgap of the well material, $E_{hh}^{w}$ is the ground state energy of the heavy hole in the well and $E_{X}>0$ the exciton binding energy. As a result, interdiffusion will directly shift the emission wavelength by two concurring effects: firstly, it reduces the confining potential. Secondly, it changes the effective masses as these depend on chemistry. Conversely, segregation describes the drive towards phase separation in a material system and can thereby increase the confining potential if, for example, two as-deposited ternary alloys de-mix into their binary compounds. It is thus clear that interdiffusion and segregation are two competing mechanism that can both change carrier confinement energies and thus influence optical and electrical properties of semiconductor quantum domains, so they need to be controlled.

This article will outline the imaging and spectroscopy techniques that can be applied in transmission electron microscopy (TEM) and scanning TEM (STEM) to measure interdiffusion and/or segregation in semiconductor nanostructures quantitatively at the (sub-) nanometre scale. It is intended as a guide to choose suitable (S)TEM-based methods and avoid common pitfalls; it is not intended as a review of related electron microscopy studies on general signal quantification or on materials other than semiconductors although related studies of ceramics, polymers and metals will sometimes encounter similar limitations as described here. As such, many of the practical examples are drawn from the author’s previous work, but care has been taken to relate these to alternative approaches by other groups.

## 2. Transmission Electron Microscopy (TEM)

There are different imaging methods that can be used in a transmission electron microscope and yield different information. The most popular ones are

(a) bright-field (BF) TEM with a small objective aperture,
(b) dark-field TEM with a small objective aperture,
(c) high-resolution TEM (HREM) with a larger objective aperture,
(d) annular dark-field scanning TEM (ADF-STEM) without any objective aperture but with a larger convergence angle of a small probe that is raster scanned over the specimen. The intensity is here registered not with a two-dimensional image detector but a ring-shaped sensitive charge integration device that produces a map of the intensity at every point. This technique necessities a scan unit but has been widely applied in the last decade because many other signals (bright-field signal, electron energy loss, characteristic X-rays produced, light emitted) can be read out simultaneously.

Electron holography, based on either the use of an electron biprism, related in-line interference methods or diffractive imaging, i.e., the reconstruction of images from sets of diffraction patterns, is extremely demanding in terms of stability of both instrument and laboratory infrastructure and is not covered here as it is deemed too specialised.

Methods (a) and (b) are mainly determined by a superposition of diffraction contrast (providing information on grain orientation) and mass-thickness contrast (providing information on scattering power that can be used to distinguish two materials if their thicknesses are similar). Methods (c) and (d) can produce lattice images with crystallographic information. Method (d) is often called ‘Z-contrast’ [2] when the collection angle is several times larger than the beam convergence angle (high-angle ADF-STEM) as then the angular dependence of the signal approaches that of Rutherford scattering of individual atoms, summed incoherently over all atoms within the same atomic column along the beam direction [3].

If the specimen is of constant thickness and only two elements can interdiffuse, i.e., in binary systems such as SiGe, or in a quasi-binary systems such as ternary InGaAs or AlGaN (where one sub-lattice is fixed), then changes of the scattering intensity can be directly interpreted in terms of chemistry. For quaternary systems, where several atomic species can interdiffuse, e.g., Al_x_Ga_y_In_1-x-y_As, Ga_x_In_1-x-_As_y_Sb_1-y_, direct spectroscopic studies are generally needed unless atomic sub-lattices can be imaged independently [4]. The same applies to co-segregation of several chemical elements, such as of Tb and O in Tb-doped AlN [5].

For uncapped quantum dots and free standing quantum nanowires whose projected thickness along the electron beam direction changes strongly with lateral position (as opposed to structures like QWs that are embedded in a matrix), this implies that image intensities depend strongly on both chemistry and local thickness so images are generally more difficult to interpret than maps from spectroscopic data.

The most common spectroscopic methods used in (S)TEM comprise

(e) energy-dispersive X-ray spectroscopy (EDXS) and related X-ray mapping,
(f) electron energy-loss spectroscopy (EELS) of ionisation core losses and their mapping in energy-filtered TEM (EFTEM),
(g) plasmon spectroscopy and mapping thereof.

Low-energy spectroscopy of intra- and interband transitions is in principle now possible by monochromated (S)TEM but the demands on spectrometer stability and alignment are extreme for low bandgap semiconductors so monochromatic studies have not really been able so far to study individual lattice defects or interfaces in semiconductors [6,7]. Effects from Cerenkov radiation [8] due to large refractive indices and the extended tails of the zero loss peak [9] make a reliable interpretation of bandgaps generally very difficult.

The (S)TEM data shown in the following sections were recorded with different instruments:

- Figures 2 and 9 in a JEOL Z3100 R005 cold field emission gun (cFEG)-STEM operated at 300 kV (beam convergence: 28 mrad; ADF inner collection angle: 62 mrad), equipped with a JED 2300 Si:Li X-ray detector with ultrathin polymer window (X-ray collection solid angle: 0.17srad);
- Figure 3 in a JEOL 2010F FEG-TEM operated at 197 kV and equipped with a Gatan Imaging Filter 2000 (beam convergence: 5 mrad; EFTEM collection angle: 37 mrad);
- Figure 5 in a JEOL 4000EX operated at 400 kV (beam convergence: ~1 mrad; BF collection angle: ~3 mrad);
- Figure 7 in a VG HB 501 cFEG-STEM at 100 kV (beam convergence: 7.6 mrad; high angle ADF inner collection angle: ~200 mrad).

All convergence and collection angles given above are semi-angles measured with respect to the optic axis. In ADF, the inner collection angle chosen is always a trade-off between signal intensity (higher at smaller angles) and ease of contrast interpretation (Z-contrast dominates at very high angles [10], strain from microstructural defects at intermediate angles [11] and diffraction contrast as well as surface strains from static atomic displacements at somewhat lower angles [12]). The precise angles chosen in experiments depend on the camera lengths available on the corresponding instruments that can yield a reasonable signal-to-noise ratio. This is a key problem if the electron beam intensity is significantly reduced by narrow monochromator slits [6,7].

## 3. Quantum Dots

**Figure 1:**
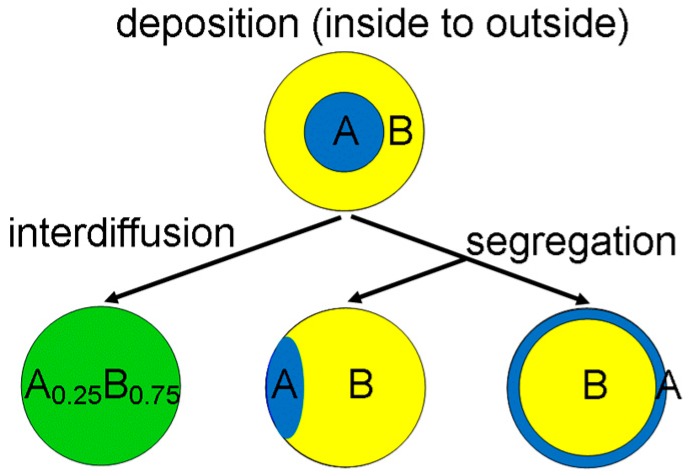
Sketch distinguishing the effects of diffusion and segregation in quantum dots. Colour is used to ascribe the chemical composition where pure A is blue (deposited first), pure B is yellow and an alloy AB is green.

Figure 1 is a schematic indicating possible changes to a core/shell system, where A-type nanoparticles were deposited first and then coated by another material B. Interdiffusion will lead to intermixing of both components, creating an alloy that will, after sufficiently long time at elevated temperature, become homogeneous (left part of Figure 1). Segregation can either stabilise the as-deposited core/shell particle (if the material deposited last has the lower free surface energy and so is more stable outside) or modify it in two ways: if A and B have similar free surface energies and a medium-high interface energy, the particle may tend towards lateral de-mixing as indicated in the lower middle part of Figure 1 (and could eventually completely separate into small A and larger B particles if their interface energy was sufficiently high). If A has a much lower free surface energy than A, however, then the core/shell structure can even become inverted, as sketched in the lower right part of Figure 1. Phase separation will thus be dominated by a competition between inner stresses due to misfit strain and surface stresses due to geometrical shape and free surface effects. It is important to note that interpretation of diffusion and/or segregation relies on knowledge of both as-deposited (initial state) and resulting structure (final state).

It is clear that electron microscopy, due to its superior resolution in combination with analytical spectroscopy, holds the key to directly observing and probing such core/shell quantum dot structures [13].

**Figure 2:**
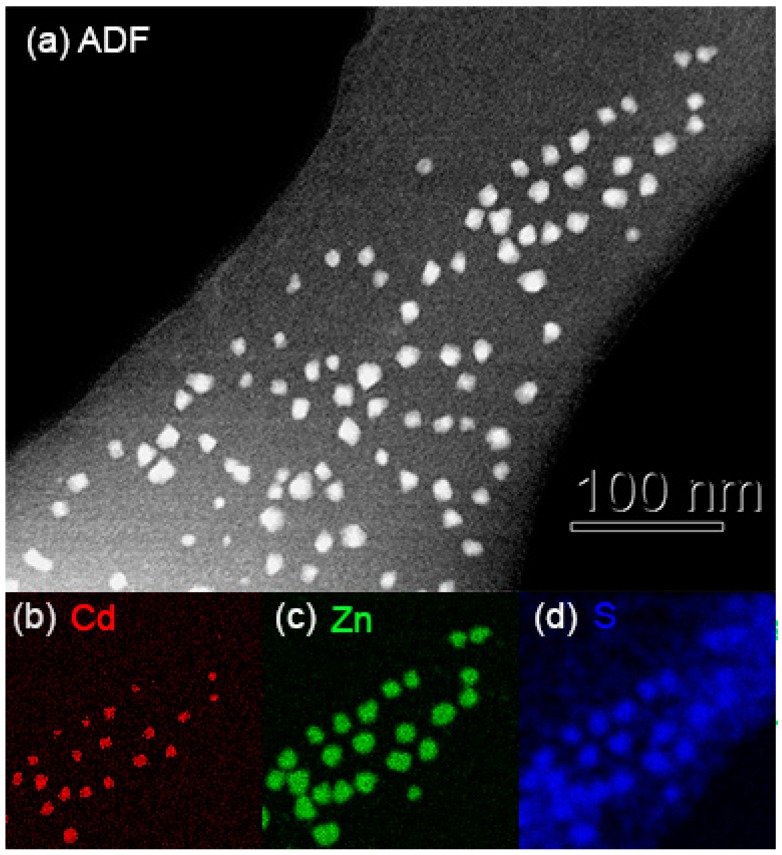
Example of CdZnS colloidal quantum dots with CdS core/ZnS shell structure (**a**) annular dark-field scanning TEM (ADF-STEM) image of a larger region and (**b**–(**d**) X-ray maps of the top right quarter of (**a**) ~180 nm wide, displaying intensities of (**b**) Cd_L_ (max = 13 counts), Zn_K_ (max = 63 counts) and S_K_ (max = 58 counts). The weak sulphur background signal in (**d**) is an artefact due to imperfect background subtraction for the sulphur K-edge at 2.3 keV; also, it suffers from tails of the neighbouring silicon K-edge at 1.8 keV as the supporting carbon film actually contained some silicon.

Figure 2 shows as an example colloidal core–shell structures of CdS/ZnS quantum dots that have undergone some intermixing due to interdiffusion where the ADF image (Figure 2a) alone does not suffice as one needs elemental maps of the distribution of characteristic X-ray emission (Figure 2b–d) to prove their brighter centres are not just thicker but also preferentially contain the heavy metal cadmium. This result is similar to what has been shown by X-ray mapping for ~10 nm wide CdSe/ZnS core/shell nanoparticles [14].

**Figure 3:**
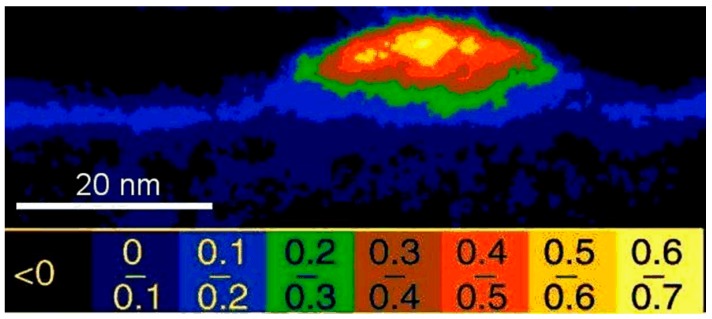
Example of uncapped epitaxial InGaAs quantum dot imaged by energy-filtered TEM (EFTEM) using both In M and Ga L edges. Indium enrichment in the centre is due to lateral segregation during growth. Reproduced from [15], with permission from American Physical Society, 4 June 2019.

Figure 3 shows a quantitative concentration map obtained from a series of energy-filtered TEM images of an uncapped InGaAs quantum dot grown epitaxially on GaAs in cross-section, indicating indium agglomeration in its centre despite a homogeneous deposition flux, which is due to lateral segregation during epitaxial growth [15,16]. If nominally pure InAs is deposited on GaAs instead, then the islands spontaneously formed on the surface are also found to be a mixed InGaAs alloy which has been explained by a combination of interdiffusion and indium desorption [17].

Local segregation of In atoms in InGaN islands [18] and nanowires [19] has also been observed by plasmon loss mapping using STEM-EELS, with a resolution on the nm-scale.

## 4. Quantum Nanowires

All structures whose length is orders of magnitude larger than their lateral widths may be called nanowires if their widths are sub-micron. If nanowires are made of semiconductors and the lateral dimensions become so small that they influence charge carrier confinement, then they may be called quantum nanowires (QNWs).

As most semiconductors crystallise in the diamond, sphalerite or wurtzite lattices, such QNWs often have prismatic shape with hexagonal cross-sections, the sidewalls being formed by either {111} planes (if cubic) or {1$\bar{1}$00} planes (if hexagonal). Key structures of technological interest involve axial (core/shell-type) as well as radial (quantum disk-like) heterostructures.

In the latter case of radial quantum disks, intensity line profiles taken in the centre of the nanowire along its long axis can be used to investigate changes in chemical composition for constant thickness, however, for axial heterostructures, profiles across the core/shell structure also involve drastic variations in thickness, so any type of imaging has to be accompanied by detailed simulations [20] to separate chemical concentration from thickness effects, while X-ray spectra generally allow a more direct interpretation of the chemistry of the cores [21].

## 5. Quantum Wells

**Figure 4:**
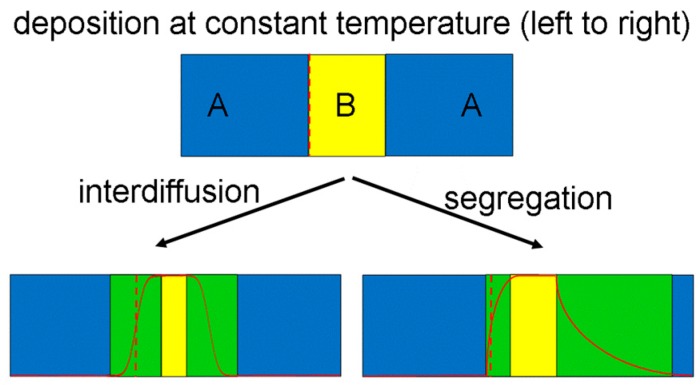
Sketch distinguishing effects of diffusion and segregation for quantum wells. Green is again used to denote a mixed alloy, and the solid red line sketches a line profile of the chemical contribution of component B. The dashed vertical red line indicates an alloy composition of A_0.5_B_0.5_.

In a QW structure A-B-A as sketched in the top of Figure 4, interdiffusion will broaden and smear the originally abrupt interfaces. This will be symmetrical if the temperature was not changed suddenly during deposition and the deposited structure held at growth temperature for roughly the same duration. Dynamical segregation of B atoms to the free surface during growth, however, will result in asymmetrical interface broadening where the long-range tail towards the free surface on the right (‘trailing or upper interface’) is often the most noticeable effect. In-situ Auger electron spectroscopy was the first technique to detect such surface segregation in semiconductors: P segregation to the Si/SiO_2_ interface was detected by depth profiling combining sputter etching with Auger analysis [22], and for SiGe the Ge signal persisted despite capping by Si for a long time during epitaxy [23]. At the same time, the position of the first-deposited ‘leading’ or bottom interface moves by exactly 1 monolayer towards the substrate due to some B atoms swapping sites with A atoms in the first deposited barrier layer. This downward shift by a single monolayer is not usually observable in any experiment because there is no sufficiently accurate marker that could be employed for reference.

It is clear that individual atomic-scale growth steps within an otherwise abrupt interface between A and B (so-called roughness) will only be visible if, firstly, the step density is low enough so that their typical distance is not smaller than the specimen thickness along the beam direction and, secondly, if the viewing direction is perpendicular to these steps—otherwise, the projection effect in transmission geometry will make it impossible to distinguish rough from diffuse interfaces [24]. This means thin samples will be required for quantitative measurements and the local specimen thickness should be determined (by electron diffraction or spectroscopy).

In analogy to the case of colloidal core–shell particles, strain can influence the formation of epitaxial QWs by, rather than incorporating deposited atoms that are too large for incorporation within a certain unit cell, driving such atoms towards the surface. In fact, misfit strain has been directly identified as the key parameter behind the Stranski–Krastanow growth in both InGaAs/GaAs (as in Figure 3) and SiGe/Si epitaxy whereby larger atomic species from a deposited wetting layer accumulate within spontaneously formed islands which, if small enough, can directly form randomly positioned QWs.

There are several different models for surface segregation during epitaxy that may be either classified as atomistic or phenomenological. Atomistic models simulate individual atomic site swaps on the surface using various atomic configurations and kinetic approximations, examples being the two-state exchange model that considers the surface monolayer and the sub-surface monolayer of a perfectly flat surface only [25,26], while the three-state exchange model considers atomic site swaps between the three topmost monolayers during epitaxy [27,28] and may also take into account the role of atomic steps, kinks and even surface reconstructions. The 3-state-model has been necessary to explain the presence of more than a single full Ge monolayer segregated on top of epitaxial SiGe [29,30]. Phenomenological models fit the extended tails of the resulting compositional profiles based on either geometric series of effective segregation ratios, *R*, between successive monolayers (giving for *n* deposited monolayers *x*_i_ = *x*_0_(1 – *R*^i^) for the i^th^ monolayer of the leading interface, 1 ≤ i ≤ *n*, and *x*_i_ = *x*_0_(1 – *R*^n^)*R*^i–n^, i > *n*, for the trailing interface) or based on exponential functions with 1/e decay lengths, *L*. Both approaches of profile fitting are equivalent because of the relationship

$$\ln R=-\frac{d_{ML}}{L}$$

where *d*_ML_ is the distance between successive monolayers measured along the growth direction [31].

**Figure 5:**
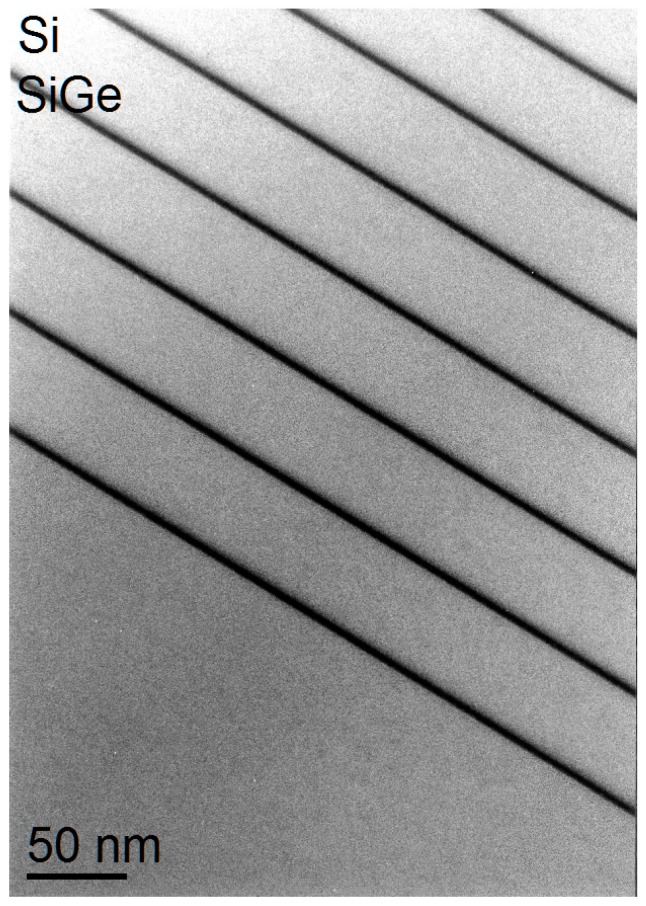
Ge surface segregation in a Si/SiGe multiple quantum well with 49 nm period length. BF TEM. Note lower interfaces appear sharp while upper interfaces are slightly blurred, proving Ge surface segregation qualitatively.

Figure 5 shows as an example multiple Si_1-x_Ge_x_/Si quantum wells, where lower and upper interfaces are visually slightly different, however, bright field imaging can introduce strong non-linearity with composition even for the relatively low Ge content of the quantum wells studied here (*x* ≈ 0.2) and so has to be treated with care for quantification [32].

High-angle ADF imaging is sometimes referred to as ‘Z-contrast’ because for sufficiently large collection angles the intensity increases with the square of the atomic number, *Z*, in agreement with Rutherford scattering [10]. For *i* atoms in the atomic column of a crystal the electron traverses, the intensity then is proportional to ∑*_i_* *Z_i_*^2^ where *Z*i is the atomic number of the *i*^th^ atom along the electron beam direction. For a simple binary alloy A*_j_*B*_k_*, we have ∑*_i_ Z_i_*^2^ = *jZ*_A_^2^ + *kZ*_B_^2^ ≠ ∑*_i_* [^1^/_2_(*Z*_A_ + Z_B_)]^2^ = *i*<*Z>*^2^, which means that in a crystal one can only work with a hypothetical average atomic number <*Z*> under certain assumptions, and the situation can be even more complicated by surface oxidation which many semiconductors are prone to [33].

**Figure 6:**
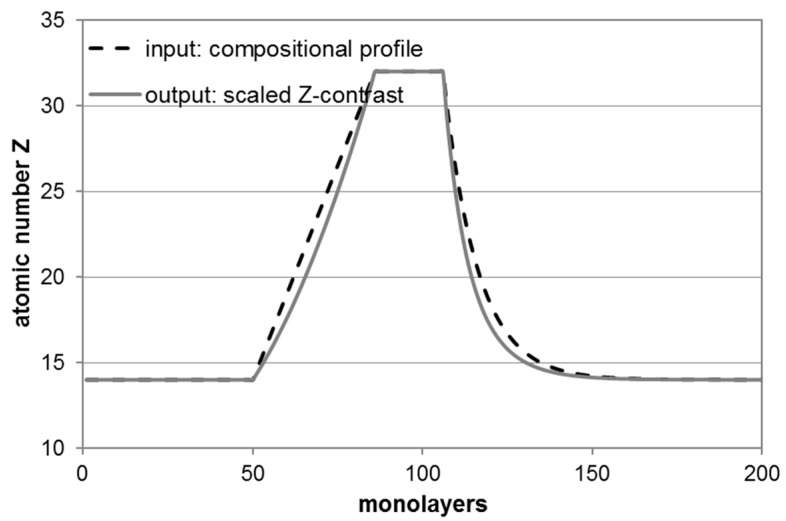
Simulation of compositional profiles through a Ge quantum well (QW) structure with (from left to right) 7 nm Si, 4.9 nm linearly graded SiGe, 2.8 nm Ge, 13 nm SiGe with exponential tail (1/e length: *L* = 1.4 nm, monolayer: *d*_ML_ = 0.14 nm).

Figure 6 demonstrates that ‘perfect *Z*-contrast’ imaging will yield an intensity profile which, when scaled to the same minimum and maximum values compared to the true underlying compositional profile, will be underestimating the concentration of the heavier atomic species (for Si_1-x_Ge_x_ with *Z*_Si_ = 14 and *Z*_Ge_ = 32 by Δ*Z* = −1.76 or Δ*x* = −0.1, i.e., 10 at%) as well as appear considerably narrower [10]. In the above example of SiGe, the full width at half maximum (FWHM) of the *Z*-contrast profile will be 40 instead of 45 ML and the full width at tenth maximum (FWTM) 69.5 instead of 75.7 ML, i.e., 5–6 ML or 0.7–0.8 nm narrower. This will be important when extracting quantitative compositional profiles. Therefore, taking the square root of an intensity profile rather than the intensity profile itself will be a much better approximation of the underlying chemical gradients as it can remove the coarse non-linearity of contrast with the chemical composition shown in Figure 6.

**Figure 7:**
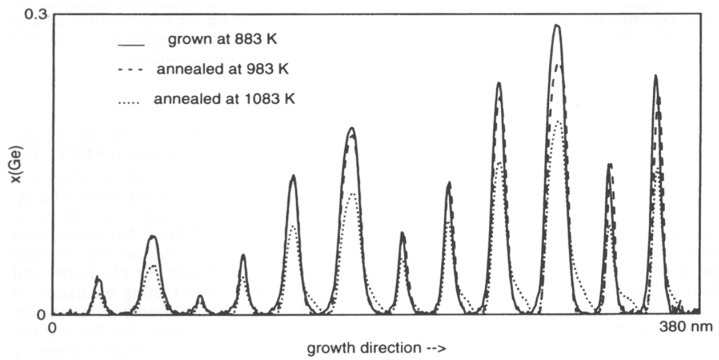
Example of interdiffusion by annealing of SiGe multilayers at 100 K above growth temperature (dashed) vs interdiffusion and some surface segregation at 200 K above growth temperature (dotted). Reproduced from [34], with permission from Scitec, 4 June 2019.

This method has been used to obtain the germanium concentration profiles in Figure 7 at ~0.5 nm resolution [34]. Repeating the quantification for two different anneal temperatures gives the possibility to separately determine diffusion constant and activation energy for interdiffusion [34,35]. For the quantum wells with high Ge content, tails towards the surface during the highest anneal temperature indicate surface segregation presumably due to in-diffusion of point defects from the surface during the anneal.

**Figure 8:**
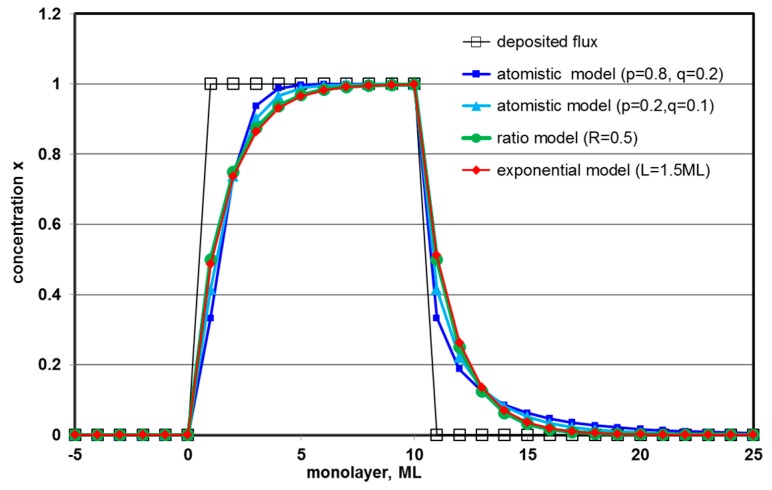
Comparison of compositional profiles from different segregation models for a quantum well nominally 10 monolayers wide.

Figure 8 compares simulations of surface segregation for a modelled quantum well only 10 monolayers thin. This model is generally valid for any (pseudo) binary system and not confined to any specific material as it only considers atomistic swaps between two different atom types with certain probabilities *p* (for jumps from sub-surface to surface monolayer) and *q* (for reverse jumps). While the deviations from the as-deposited square box-shaped profile are obvious for all simulations, differences between them are actually rather small and typically confined to the concentration values of only two intermediate monolayers on either side, indicating that not only atomic resolution but also high fitting accuracy for each monolayer would be required to distinguish them. Hence, to measure segregation ratios or lengths from experimental data it will generally be more reliable to use extended fit ranges that include many data points (i.e., modelling the long tail components) rather than attempting to fit a model to a few data points only.

Methods to extract and analyse such compositional profiles of semiconductor QWs with single monolayer (in the diamond lattice) or bilayer (in the sphalerite or wurtzite lattices) spatial resolution all involve measuring either the amplitudes or the positions of lattice fringes. Amplitude measurements include high-resolution lattice imaging with chemically sensitive (002) type reflections in sphalerite lattices at certain specimen thicknesses, e.g., (Al)GaAs [24], (In)AlAs [36], (In)GaAs [37], Ga(Sb)As [38], Ga(In,N)As [39] and (Cd)ZnSe [40]. ADF imaging can alternatively be used to map the intensity of atomic columns from which the chemical composition in simple binary and ternary compound semiconductor (pseudo-binary) systems, such as InGaN, can be inferred if the thickness influence is taken into account [41,42].

The positions of lattice fringes can be determined and their displacements be interpreted in terms of lattice strain [43] if surface relaxation in the thin foil specimen remains negligible and if the chemical composition either does not change at all [44] or changes by so small an amount that it does not visibly change the image pattern [45].

One key problem, however, is that composition and strain are inter-related in strained layers and so will influence each other. Lattice fringe intensities and displacements can either be fitted together in high-resolution electron microscopy [46,47] or electron holography methods can be applied to the same effect [48].

**Figure 9:**
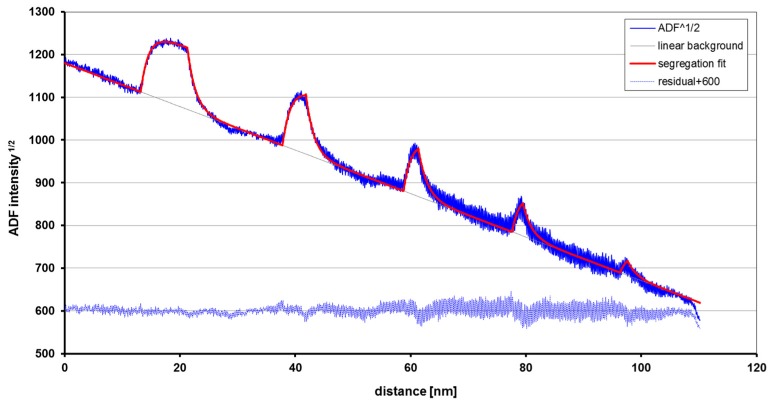
Al surface segregation from AlGaN into GaN Qws from the square root of the HA-ADF STEM profile intensity. The fit is obtained after linear background extrapolation and subtraction, modelling all QW profiles as exponential functions with 1/e decay lengths of 1.34 ± 0.04 nm for the leading and 1.45 ± 0.05 nm for the trailing edges (upper interfaces, towards the right). *d*_ML_ = *c*/2 = 0.26 nm. The linear gradient is due to the wedge shape geometry of the specimen, and the high-frequency oscillations visible towards the thinner end are actually due to *c*/2 lattice planes. Reproduced from [42], with permission from Springer Nature, 4 June 2019.

Figure 9 shows that the profile of a set of 5 quantum wells, in this case of AlGaN, can be well fitted with only three parameters: one for the peak concentration (which is not actually reached in the cases of the thinnest quantum wells) and decay lengths for each interface side, yielding highly reproducible measurements for all quantum wells [42]. The resulting segregation ratios resulting from the numerical values of the decay length stated in the figure caption would be 82.4 ± 0.5% for the leading and 83.6 ± 0.5% for the trailing interfaces.

## 6. Segregation at Grain Boundaries, Interfaces, Defects and Surfaces

There are some issues particular to segregation in semiconductors:

(a) Grain boundary segregation can unintentionally and directly lead to the formation of extremely thin quantum wells, down to fractions of monolayers, where imaging approaches can fail even with the best electron microscopes as it is chemical sensitivity and accuracy in measuring chemical profiles that count rather than spatial resolution. Interfaces that appear atomically smooth in lattice images may in fact be chemically graded if the interference pattern is not sufficiently sensitive to those gradients, cf. the case of Ca segregation [49] vs. Ba segregation [50] at SrTiO_3_ on (La,Ca)MnO_3_ interfaces. Conversely, strained interfaces can also appear diffuse in imaging when they are actually abrupt, due to long range strain components interfering with the contrast in bright field [51,52] as well as medium-angle dark field [12].
(b) For interfaces and grain boundaries thinner than a unit cell or a fraction of a monolayer, a method originally implemented in TEM [53] and later also STEM [54] has been successful in measuring highly accurately the effective chemical width of fractions of monolayers in many material systems, most recently for Ge/Si [28] and InAs/GaAs [55,56]. This approach uses a plot of atomic ratio measured as a function of scan window size perpendicular to the interface or defect in question. If the spatial resolution is sufficient and the sample does not damage at high electron dose, then atomic resolution images of grain boundaries, ideally correlated with simultaneously acquired EELS, can sometimes directly reveal the positions to which atoms segregate, e.g., heavy metal dopants at interfaces in polycrystalline Si_3_N_4_ [57].
(c) Atomic segregation to lattice defects or surfaces can directly form quantum nanowires or quantum dots, depending on the extension of the defects structure, and both surface segregation and interdiffusion can modify the chemistry of such quantum domain structures further. While many spectroscopic methods in (S)TEM reach only nm-scale rather than lattice resolution, this is often sufficient to detect such surface layers, e.g., thin surface layers of phosphides used for passivation of the sidewalls in GaAs-based quantum nanowires (as in the supporting information to [58]). EDXS point analysis proved Al segregation to dislocation cores in AlGaN [59]. Both ADF imaging and X-ray line scans have been combined in [60] to detect indium segregation to the sidewalls of V-shaped pyramidal surface defects in (In)AlN. ADF and EELS were used to detect oxygen segregation to screw dislocations in GaN [61].

## 7. Conclusions

Strategies have been discussed to detect, distinguish and measure interdiffusion and segregation in semiconductor quantum domain systems using transmission electron microscopy.

For quantum dots, compositional mapping from spectroscopic methods such as STEM-EDXS or EFTEM have been demonstrated to be more reliable than pure imaging approaches, due to problems related to local variations of the specimen thickness that influences the contrast in any type of imaging.

For quantum wells viewed in cross-section, image profiles can be quantified more easily, e.g., in ADF-STEM by calculating the square root of the intensity before fitting any model to a compositional profile. Segregation ratios and segregation lengths can easily be converted into each other as their models are equivalent. Fitting models to very narrow single quantum wells may be prone to errors if the data points contain some degree of noise, while fitting the tails over extended ranges is more reliable.

If chemical profiles of the same structures annealed at different temperatures are compared, it is possible to separate the diffusivities measured into diffusion constants and activation energies for interdiffusion.
